# Retinal-input-induced epigenetic dynamics in the developing mouse dorsal lateral geniculate nucleus

**DOI:** 10.1186/s13072-019-0257-x

**Published:** 2019-02-14

**Authors:** Jianlin He, Xiguang Xu, Aboozar Monavarfeshani, Sharmi Banerjee, Michael A. Fox, Hehuang Xie

**Affiliations:** 1 Biocomplexity Institute of Virginia Tech, Blacksburg, VA 24061 USA; 2 Department of Biological Sciences, Virginia Tech, Blacksburg, VA 24061 USA; 3 Developmental and Translational Neurobiology Center, Fralin Biomedical Research Institute at Virginia Tech Carilion, Roanoke, VA 24016 USA; 4 Bradley Department of Electrical Engineering, Virginia Tech, Blacksburg, VA 24061 USA; 5 Department of Pediatrics, Virginia Tech Carilion School of Medicine, Roanoke, VA 24016 USA; 6 Department of Biomedical Sciences and Pathobiology, Virginia-Maryland College of Veterinary Medicine, Blacksburg, VA 24061 USA

**Keywords:** DNA methylation, Dorsal lateral geniculate nucleus, Transcription factor, *Math5*, Eye opening

## Abstract

DNA methylation plays important roles in the regulation of nervous system development and in cellular responses to environmental stimuli such as light-derived signals. Despite great efforts in understanding the maturation and refinement of visual circuits, we lack a clear understanding of how changes in DNA methylation correlate with visual activity in the developing subcortical visual system, such as in the dorsal lateral geniculate nucleus (dLGN), the main retino-recipient region in the dorsal thalamus. Here, we explored epigenetic dynamics underlying dLGN development at ages before and after eye opening in wild-type mice and mutant mice in which retinal ganglion cells fail to form. We observed that development-related epigenetic changes tend to co-localize together on functional genomic regions critical for regulating gene expression, while retinal-input-induced epigenetic changes are enriched on repetitive elements. Enhancers identified in neurons are prone to methylation dynamics during development, and activity-induced enhancers are associated with retinal-input-induced epigenetic changes. Intriguingly, the binding motifs of activity-dependent transcription factors, including EGR1 and members of MEF2 family, are enriched in the genomic regions with epigenetic aberrations in dLGN tissues of mutant mice lacking retinal inputs. Overall, our study sheds new light on the epigenetic regulatory mechanisms underlying the role of retinal inputs on the development of mouse dLGN.

**Electronic supplementary material:**

The online version of this article (10.1186/s13072-019-0257-x) contains supplementary material, which is available to authorized users.

## Introduction

DNA methylation is a lasting epigenetic mark for repressive chromatin domains. The addition or removal of methyl groups on DNA is an important way to achieve refined regulation of gene expression. DNA methylation dynamics have significant functional effects on cell specification and differentiation during tissue development and the environmental adaptation of higher-order living organisms. Recent studies indicate that DNA methylation dynamics contribute to the development of visual circuits in the brain, and malfunctions of DNA methylation machinery lead to the developmental defects in this system [1, 2]. Likewise, DNA demethylation on genes involved in the *Notch* and *Wnt* pathways has been shown to be essential for the differentiation and morphogenesis of retinal ganglion cells (RGCs), the projection neurons of the retina [3]. Moreover, visual experience leads to epigenetic reprogramming on a set of plasticity genes in developing visual cortex [1–3]. However, we lack any knowledge on how visual input, activity or experience alters the epigenome of the dorsal lateral geniculate nucleus (dLGN), a retino-recipient thalamic nucleus that relays image-forming visual information from retina to primary visual cortex.

 The formation and maturation of visual circuits in rodent dLGN takes place over a protracted developmental period that begins embryonically and continues for several weeks after birth, when light-derived visual activity contributes to the refinement and function of visual circuits [4–6]. In rodents, retinal ganglion cells are generated at mid-gestation, their axons extend out of the retina and eye by E14, and the first retinal axons invade and innervate dLGN at E16 [7, 8]. During the first postnatal week, retinal projections continue to innervate dLGN and begin to segregate into eye-specific domains [9, 10]. Retinal synapses in dLGN (termed retinogeniculate or RG synapses) emerge during the first postnatal week of mouse development [11], but these immature synapses lack the ultrastructural features that are characteristic of adult retinogeniculate synapses [12, 13]. Activity-dependent refinement of these connections begins in the first postnatal week of development and continues beyond eye opening, which is at postnatal day 12 in mice [14, 15].

During the first three postnatal weeks not only are there changes in retinal inputs and retinogeniculate synapses in dLGN, but many other changes are ongoing in this region of visual thalamus. In fact, the size of the dLGN expands threefold in rodents during this period [16]. Two main classes of neurons exist in mouse dLGN, principle thalamocortical (TC) relay cells and a small population of local inhibitory interneurons, both of which are directly innervated by retinal axons [10, 14, 17]. TC relay cells have already acquired their morphology at P6 and grow extensively after P6. Interneurons are recruited into the dLGN from other thalamic and tectal regions postnatally and begin to form inhibitory synapses by eye opening [11, 12]. During this period, a large number of non-retinal inputs also innervate neurons in dLGN. These inputs are mainly derived from neurons residing in superior colliculus, brainstem, thalamic reticular nucleus and layer VI of primary visual cortex [4–6].

Retinal inputs and activity (in the form of either spontaneous activity or light-evoked activity) play critical roles in shaping dLGN development. First, spontaneous activity in RGCs contributes to activity-dependent refinement of retinogeniculate synapses and the formation of retinotopic maps [9, 10]. Second, retinal inputs play instructive roles in the timing of dLGN innervation by a number of non-retinal inputs, including corticogeniculate inputs [18–20]. Third, retinal inputs and retinal activity are required for the normal recruitment of local inhibitory interneurons into dLGN from their thalamic and tectal sources [21, 22]. Finally, retinal inputs are required for the normal developmental remodeling of thalamocortical relay cells [16]. Here, we set out to address how retinal inputs influence so many aspects of dLGN development. We specially sought to understand if innervation of TC relay cells by RGC axons or activity transmitted through those axons altered the dLGN methylome. Rather than assessing this in animals whose retinogeniculate connections have been anatomically or functionally disrupted postnatally, we examined methylome and transcriptome changes in the mutant mice lacking the *Math5* gene (also known as *Atoh7*). *Math5* is a basic helix-loop-helix (bHLH) transcription factor that is expressed in retinal progenitors starting at E11 and is essential for the generation of RGCs [16, 23, 24]. Importantly, *Math5* is largely absent from dLGN [16]. Mice lacking *Math5* (Math5KO mice) are fully viable but lack RGCs and all retinofugal projections, even at early embryonic ages [18, 20, 25]. Thus, these mice are an ideal model to study the role of retinal input in epigenetic dynamics of the developing dLGN [16, 24, 25].

In this study, we characterized the dLGN methylomes and transcriptomes before and after eye opening in Math5KO mice and wild-type control mice. We obtained global methylation profiles to identify normal changes during development and aberrant methylation markers associated with the loss of retinal input. Integrating with brain methylome data and ChIPseq data for histone marks and transcription factors, we predicted key transcription factors critical for epigenetic programming of dLGN cells.

## Results

### Genome distribution of dynamic methylated loci in dLGN during development and in response to retinal input

**Fig. 1:**
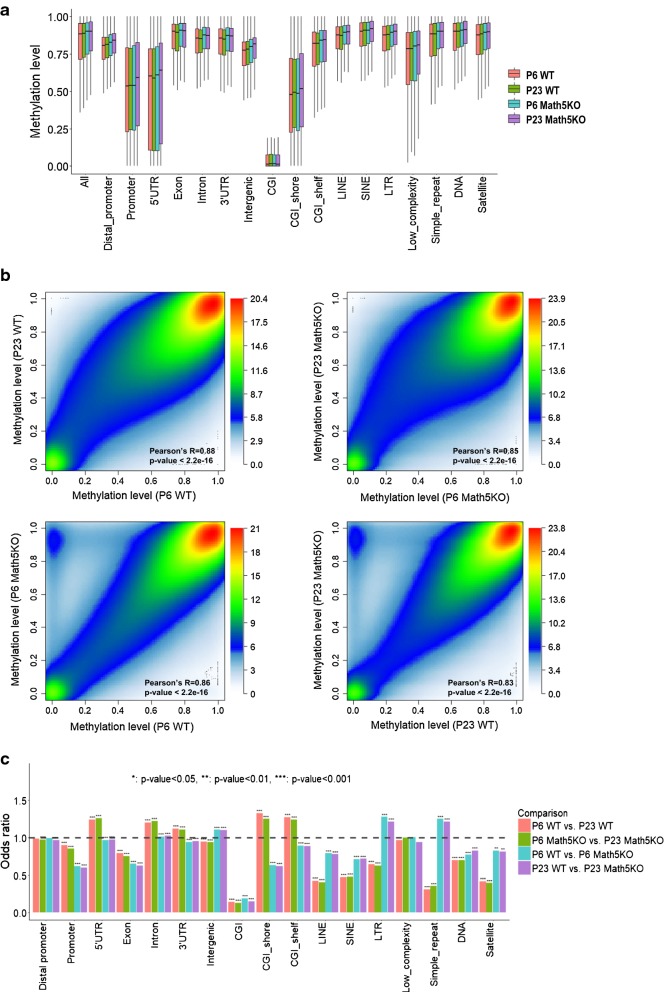
Whole-genome bisulfite sequencing of dLGN tissues dissected from wild-type (WT) and Math5 knockout (Math5KO) mice at P6 and P23. **a** Methylation levels of diverse genomic compartments. Promoter is defined as the upstream 2 kb of transcriptional start site (TSS) to TSS. Distal promoter refers to from 10 to 2 kb upstream of TSS. **b** Pairwise comparisons of four dLGN WGBS libraries. The color from white to red represents the density of CpG sites from low to high. **c** The enrichment of DMS sites on genomic compartments. The enrichment is relative to all CpG sites as control. Odds ratio and *p* value were calculated using Fisher exact test

To explore dLGN methylation dynamics during development and upon neuronal activity, we performed whole-genome bisulfite sequencing on dLGN tissues dissected from wild-type (WT) and *Math5* knockout (Math5KO) mice at postnatal day 6 (P6) and P23 ages corresponding to before eye opening when RG connections are still forming and refining and after eye opening when activity-dependent refinement of the RG circuit is largely completed. For each methylome, we generated around 631 million read pairs with an average of 343 million read pairs uniquely mapped to the mouse reference genome (Additional file 1: Table S1A). For these four methylomes, approximately 46.3 to 56.2% of all CpG sites were covered by at least 10 reads (Additional file 2: Fig. S1). Similar to mouse brain or neuron methylomes reported in previous studies [26, 27], low methylation was observed at the CGI, CGI shore, promoters and 5’UTR, but other genomic regions including gene body and repetitive elements are heavily methylated (Fig. 1a). In addition, DNA methylation across all CpG sites shows bimodal distributions with 36.6% hypermethylated CpG sites (methylation level ≥ 0.8) and 3.2% hypomethylated CpG sites (methylation level ≤ 0.2) (Additional file 2: Fig. S2).

We observed significant correlations among these four samples with Pearson’s R above 0.82 for any of the four pairwise comparisons (Fig. 1b): including development-related comparisons (P6 WT vs P23 WT and P6 Math5KO *vs* P23 Math5KO) and genotype (or retinal-input)-related (P6 WT vs P6 Math5KO and P23 WT vs P23 Math5KO). From P6 to P23, global DNA methylation tilted slightly toward hyper-methylation in both Math5KO and WT samples. Although the methylation profiles in KO mice largely resembled those of wild-type controls, a number of CpG sites appeared heavily methylated in KO mice but hypomethylated in WT mice. To determine differentially methylated sites (DMSs), we performed Fisher exact test with FDR control using a sequential permutation method [26] and identified over two hundred thousand DMSs for the aforementioned four comparisons (Additional file 1: Table S1B). Venn diagram demonstrates that only 126 CpG sites were found to be shared by all four lists of DMSs (Additional file 2: Fig. S3). However, substantial overlaps were observed between the lists with identical genotype or at the same developmental stage. In particularly, approximately 55.9% of DMSs identified for P6 WT versus P6 Math5KO were found in the DMS list for the comparison between P23 WT versus P23 Math5KO.

We next determined the distribution of DMSs across the genome. Interestingly, DMSs in all four lists were significant depleted from CpG islands, promoters and exons (Fig. 1c). It suggests that, irrespective of the presence of retinal inputs, most of these functional domains maintain their methylation profiles in dLGN before and after eye opening. On the other hand, in either Math5KO or WT mice, development-related DMSs are significantly enriched in the 5′-UTR, CCI shore and CGI shelf. For instance, DMSs identified in P6 WT vs. P23 WT comparison are significantly enriched on the 5′UTR (odd ratio: OR = 1.24, *p* value = 2.44e−209), CGI shore (OR = 1.33, *p* value = 2.86e−207) and CGI shelf (OR = 1.27, *p* value = 8.82e−110). In contrast, the retinal-input-induced DMSs are significantly enriched in long terminal repeats (LTR) and simple repeats but depleted in either short or long interspersed nuclear elements (SINE or LINE repeats). LTR elements are excluded from gene-rich regions [28], and the methylation level of CG-rich LTRs is highly dynamic during differentiation at early embryonic stages [29]. SINE repeats frequently co-localize with actively transcribed genes, and the methylation statuses of some SINEs have been associated with tumor aggressiveness and relapse [30]. The differences in the genome distribution of DMSs indicate that the methylation changes during dLGN development and upon neuronal activity could have a very different impact on genome function. Since the 5′-UTR, CGI shore and CGI shelf are likely to host distal regulatory elements, the development-related DMSs may have a broader functional impact on the regulation of gene expression compared with the retinal-input-induced DMSs.

**Fig. 2:**
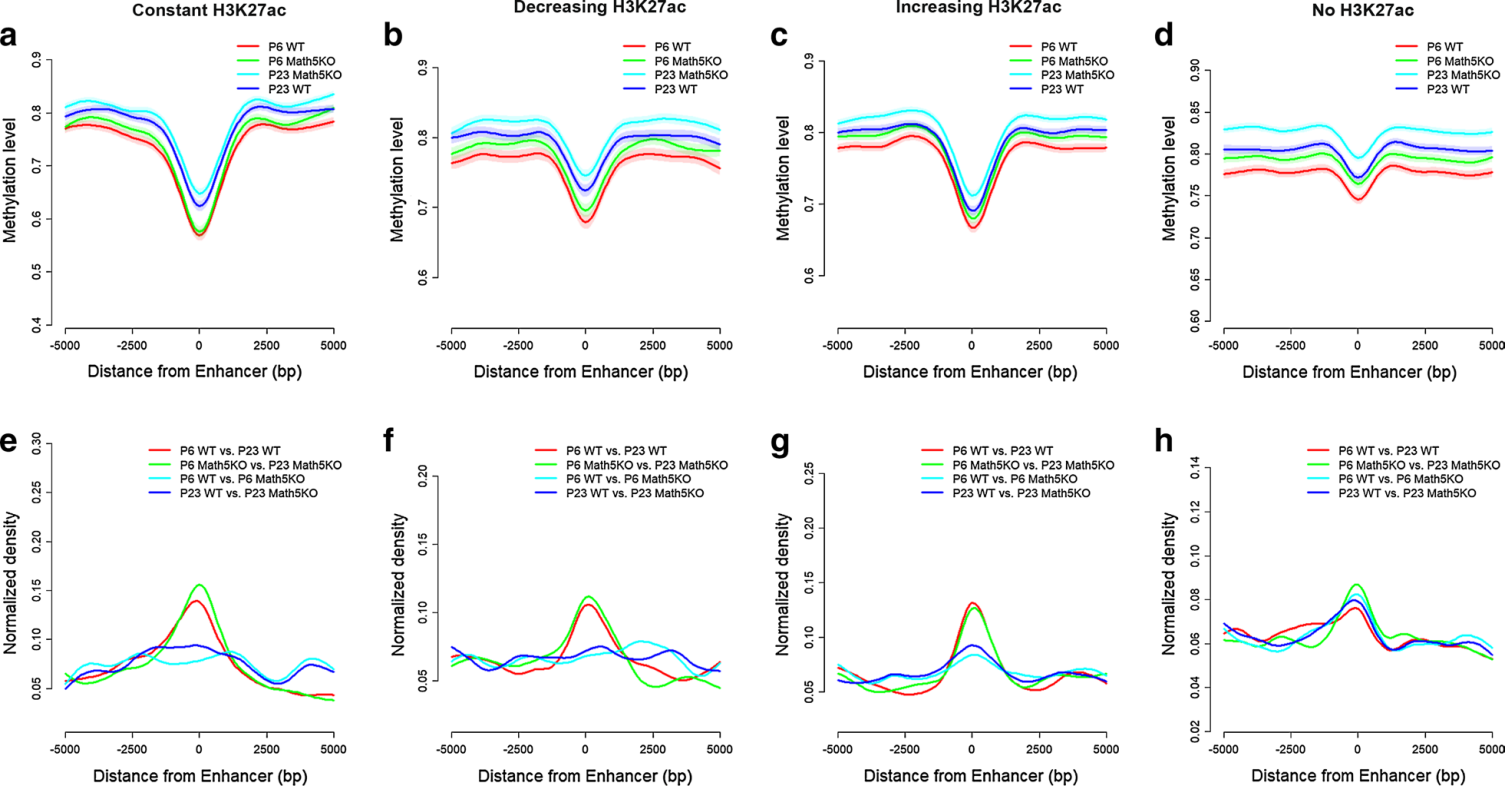
Methylation levels (**a**–**d**) of and the enrichment of DMS sites (**e**–**h**) on four types of neuronal activity-associated enhancers. Neuronal activity-associated enhancers with CBP/H3K4me1 marks were classified into four groups, according to the changes of H3K27ac upon KCl stimulation: constant H3K27ac, increasing H3K27ac, decreasing H3K27ac and no H3K27ac. In **a**–**d**, confidence band for each line represents the average of methylation level ± S.E.M. The *y*-axis in **a**–**d** denotes the methylation level, and the *y*-axis in **e**, **f** denotes the frequency of DMSs identified in pairwise comparisons surrounding enhancers normalized to the genome average

Since visual experience is tightly linked to neuronal activation, we made use of available ChIPseq datasets that identified enhancer histone marks generated from primary cultured neurons in response to KCl stimulation [31, 32]. In the previous study, 11,830 CBP/H3K4me1-enriched loci were defined as neuronal activity-associated enhancers by excluding the ones adjacent to annotated TSSs [31]. Four groups of enhancers were determined based on the dynamic changes in H3K27ac peak before and after membrane depolarization [32]: Con-H3K27ac (Con, constant; *n* = 800), Dec-H3K27ac (Dec, decreasing; *n* = 738), Inc-H3K27ac (Inc, increasing; *n* = 1468), and No-H3K27ac (premarked by H3K4me1 but in the absent of H3K27ac, *n* = 1886). We integrated the dLGN methylome data with these neuronal activity-associated enhancers to determine the average methylation levels flanking enhancers (Fig. 2a–d). For all four kinds of enhancers, DNA methylation levels consistently decrease approaching the centers of enhancers. Compared to the other three dLGN methylomes, the methylome from P23 Math5KO mice exhibits higher methylation levels across entire enhancer regions as observed in genome wide (Fig. 1a). This suggests that, in the dLGN tissue lacking retinal inputs, the enhancers identified in neurons are more methylated, and thus likely to be less active. In addition, we observed that the frequencies of DMSs identified for all comparisons increase approaching the center of enhancers (Fig. 2e–h). More specifically, development-related DMSs are enriched in all kinds of enhancers and the retinal-input-induced DMSs are enriched in the centers of two types of enhancers: the neural activity related enhancers with increased H3K27ac signal upon KCl stimulation and the poised enhancers with H3K4me1 marks that lack H3K27ac marks. These results indicate that the enhancers identified in neurons are exceeding prone to carry DNA methylation changes during dLGN development compared with that observed across the entire genome. In addition, poised enhancers in neurons and neuronal activity-associated enhances are likely to have methylation aberrations in dLGN derived from Math5KO mice.

### Non-CpG methylation and its correlation with gene expression in dLGN during development and in response to retinal input

We observed increased mCH level in dLGN methylomes during development as reported previously during frontal cortex brain development [26]. To show the relationship between mCH and gene expression, we performed RNAseq for both WT and Math5KO dLGN at four time points including P3, P7, P14 and P23 (Additional file 3: Table S2). According to gene expression level, we partitioned genes into four groups: not expressed, the bottom one-third expressed, the median one-third expressed and the top one-third expressed (Additional file 2: Fig. S4). At early postnatal developmental stage, most genes have a low mCH across generic regions but the top expressed genes contain high mCH near their transcription end sites (TES). At P23, the mCH levels at TSSs of the top expressed genes show much lower methylation levels compared with those of three other categories of genes. For those top expressed genes, mCH levels are high before TES but drop after TES.

Using pairwise comparisons of RNAseq data, we found that differentially expressed genes during development are largely overlapped in WT and Math5KO dLGN. Common sets of 463 upregulated and 554 downregulated genes from P3 to P23 were identified in both WT and Math5KO (Additional file 2: Fig. S5A, B). On the other hand, the lists of retinal-input-induced differentially expressed genes are much smaller and no gene was identified to be overlapped in pairwise comparisons between WT and Math5KO dLGN across all four time points (Additional file 2: Fig. S5C, D). We further explored the links between changes in non-CpG methylation and gene expression for aforementioned common sets of differentially expressed genes in development. Similar to all genes (Additional file 2: Fig. S4), we observed a global increase in mCG methylation from 100 kb upstream to 100 kb downstream of genes (Additional file 2: Fig. S6). For both upregulated and downregulated genes during development, a small drop in mCH methylation was found in genomic regions surrounding TSSs. Interestingly, genes upregulated during development show increased mCH methylation level at TESs compared with downregulated ones in all four dLGN methylomes, and such difference in mCH methylation is more prominent in Math5KO dLGN methylomes.

### Determination of differentially methylated regions (DMRs) and their associations with cell-type-specific methylation

To determine the genomic loci associated with methylation dynamics, we adopted the procedure described in a previous study [26] to merge neighboring DMSs into differentially methylation regions (DMRs). A DMR defined in this study meets three criteria: (1) it must contain at least five DMSs within a 500 bp window; (2) at least 80% of DMSs within a candidate DMR show methylation changes in the same direction; (3) at least 80% of CpG sites in a candidate DMR have methylation level changes larger than or equal to 0.1 and in the same direction as the majority of DMSs in the same DMR. Although the numbers of DMSs are comparable among the four pairwise comparisons, we identified five to ten times more DMRs associated with dLGN development than those associated with the loss of retinal input at any given age. Similarly, compared with the retinal-input-induced epigenetic changes, five to eight times more genes were determined to have development-related DMRs in the gene body or within 10 kb upstream from their transcriptional starting sites. Development-related DMSs tend to co-localize together in the genome, and the CpG sites within a candidate DMR share similar methylation profiles. On the other hand, retinal-input-induced epigenetic changes distributed sparsely across the genome and neighboring CpG sites are with higher methylation variation. Thus, starting with similar numbers of DMSs, development-related epigenetic changes end with much more DMRs, while much less retinal-input-induced DMR regions survived the filters for DMR determination using the aforementioned criteria 1 and 3 (Additional file 1: Table S1B). Since P23 Math5KO show increased DNA methylation in promoters (Fig. 1a), we determined 61 genes whose promoters (from TSSs to 2 kb upstream of TSSs) overlapped with DMRs showing methylation increased in P23 Math5KO. We further determined gene expression profiles (Additional file 2: Fig. S7A) and provided scatter plot of gene expression between P23 WT and P23 Math5KO (Additional file 2: Fig. S7B). Unfortunately, we were not able to generalize a rule to simply correlate gene expression to the DMR methylation at promoters of these 61 genes.

**Fig. 3:**
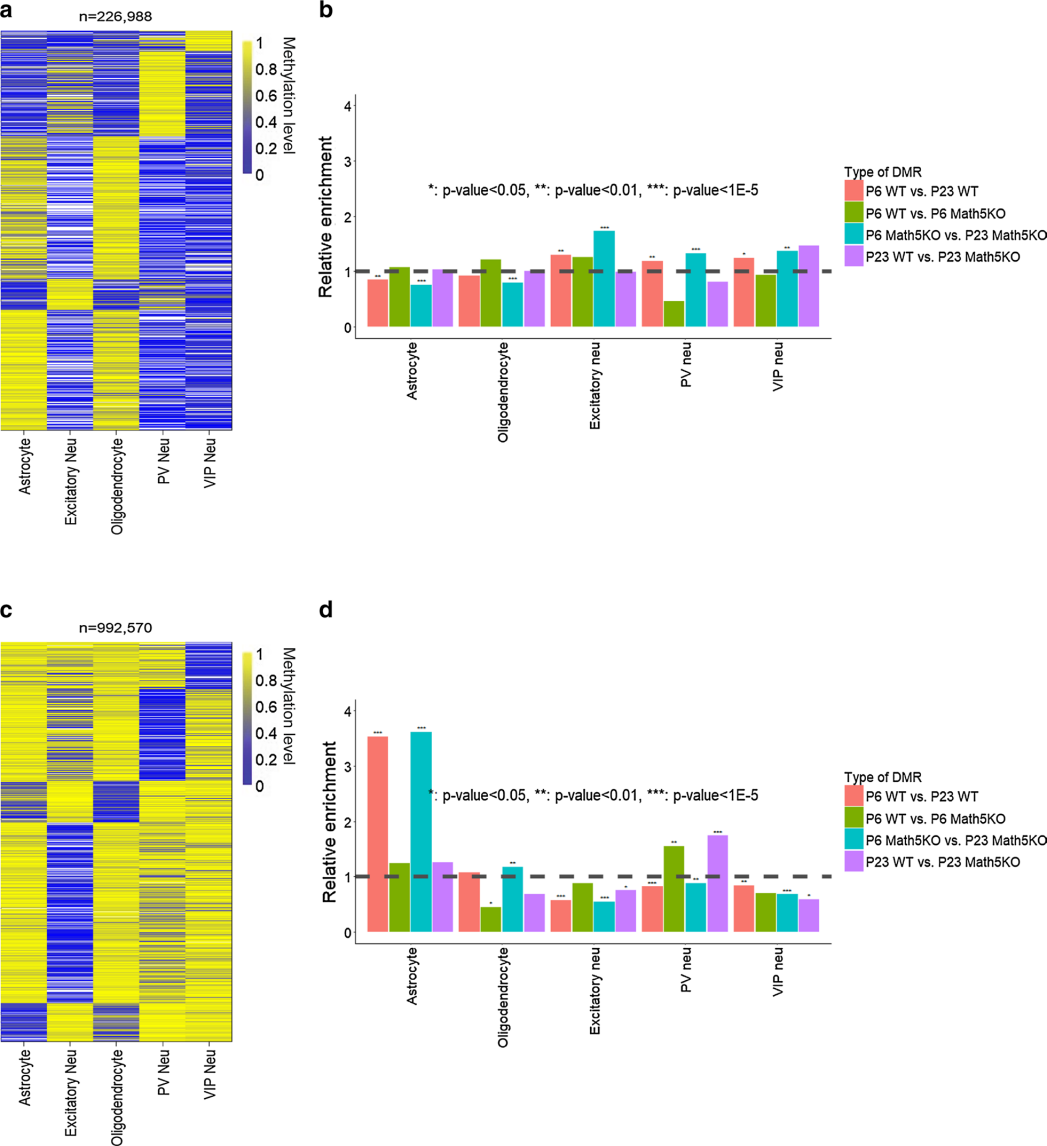
Identification of cell-type-specific **a**, **b** hyper- and **c**, **d** hypo-CpG sites and the enrichment of DMRs on cell-type specifically methylated sites (**c**, **d**). In **a**, **c** blue color denotes hypomethylation, yellow color denotes hyper-methylation, and white color denotes missing data. In **b**, **d** the control is the entire pool of cell-type-specific sites

To examine whether DMR identified in this study would reflect cell-type-specific methylation profiles, we re-analyzed brain methylomes generated for five neural cell types including excitatory neurons, two types of inhibitory neurons (PV and VIP neurons), astrocytes and oligodendrocytes [27, 33]. For each cell type, we first identified their specific methylated CpG sites, hyper-CpG sites and hypo-CpG sites (Fig. 3a, c). Compared with three types of neurons, astrocytes and oligodendrocytes share similar methylation profiles across entire genome and on their cell-type-specific methylated sites as well. We next determined the enrichment of differentially methylated regions identified for dLGN methylomes on the cell-type-specific methylated sites (Fig. 3b, d). We observed that all kinds of DMRs are depleted from the genomic sites hypomethylated in excitatory neurons. Development-related DMRs show over threefold enrichment in the genomic sites hypomethylated in astrocytes. In contrast, retinal-input-induced DMRs show enrichment in genomic sites hypomethylated in inhibitory neurons. Although the enrichment of DMRs in hypermethylated sites for all cell types are less prominent, the development-related DMRs are with increased representation in the hypermethylated sites in all three types of neurons. These results suggest that during dLGN development, demethylation may occur on a number of regulatory sites critical for glial cells. On the other hand, retinal-input-induced methylation changes may occur in neurons, particularly in the inhibitory neurons.

### Epigenetic regulated genes in dLGN during development and in response to retinal input

**Fig. 4:**
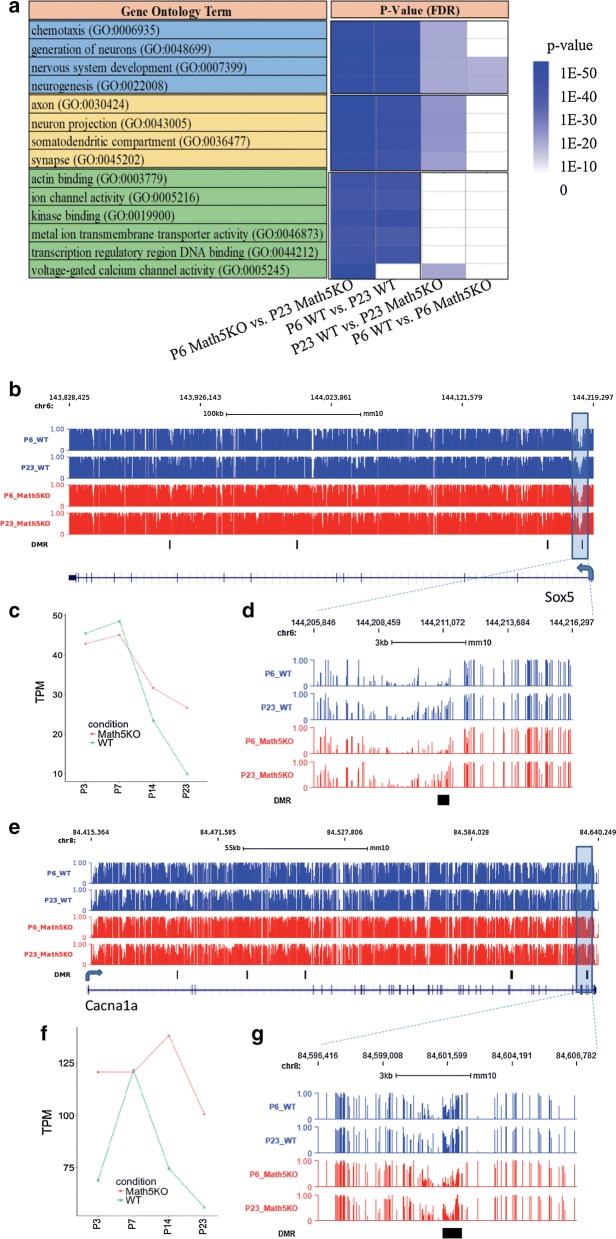
GO enrichment (**a**) of genes associated with dLGN epigenetic changes and gene expression and DNA methylation profiles for Sox5 (**b**–**d**) and CACNA1A (**e**–**g**). The genes associated with dLGN methylation changes are with DMRs in gene body or within 10 kb upstream from their transcriptional starting sites. **a** The color bar from white to dark blue denotes the significance of GO term from low to high. The genomic loci for **b** and **e** are chr6:143,828,425-144,219,297 and chr8:84,415,364-84,640,249, respectively

We next generated four lists of genes with DMRs in gene body or within 10 kb upstream from transcriptional starting sites and performed gene ontology analysis using PANTHER software [34]. Not surprisingly, we found that development-related DMRs are rich in genes with GO terms such as “nervous system development” and “neurogenesis” (Fig. 4a and Additional file 4: Table S3). In addition, the localization of these gene products is in cellular components such as “neuron projection,” “synapse” and “somatodendritic compartment.” Functionally, the genes that are epigenetically regulated during dLGN development from P6 to P23 play important roles in ion channel activity and actin binding and kinase binding, which are known to be critical for proper neuronal function [35, 36]. Among the short list of GO terms enriched for genes with epigenetic aberrations in Math5KO dLGN, “nervous system development” was found but with a much less statistical significance. For instance, the *p* value of GO term enrichment for “nervous system development (GO:0007399)” is 3.06E−51 in the P6 WT versus P23 WT comparison, while it is only 4.78E−2 in P6 WT versus P6 Math5KO.

Interestingly, we found a significant number of transcription factors that are epigenetically regulated in dLGN from P6 to P23. The enrichment *p* values for the GO term “transcription regulatory region DNA binding (GO:0044212)” are 7.66E−16 and 6.76E−11 for WT and KO development, respectively. Intriguingly, this GO term is not enriched in the comparisons between Math5KO and WT. This further confirmed that the developmental transcriptional regulatory networks remain largely intact in Math5KO mice. To examine the correlations between gene expression and DNA methylation for the selected DMRs, we made use of public methylome data for developing brains, including the forebrains at embryonic stages and the frontal cortices from 1 week to 22 month [26]. We first examined the methylation profiles of DMRs identified in the forebrains at embryonic stages and the frontal cortices after birth. Interestingly, for development-related DMRs, we observed substantial methylation increase in frontal cortices between the first and second week after birth (Additional file 2: Fig. S8). This suggests that, for some development-related DMRs identified in dLGN, their methylation dynamics may be shared by other regions as well in developing brains. We next focused on development-related DMRs identified in transcription factors and observed that DNA methylation increased during development for DMRs within Lhx2, Sox5 and Sox6 genes. In addition, the expression levels of these genes decrease from P7 to P23 during dLGN development (Fig. 4b, c and Additional file 2: Fig. S9A–C).

In searching for epigenetic aberrations in Math5KO mice, we found that seven genes involved in “voltage-gated calcium channel activity” (GO:0005245) may be linked to visual experience for which retinal inputs are required. This GO term is significantly enriched in Math5KO versus WT comparison at P23 stage but not at P6 stage. Notably, these genes also show methylation changes as well during normal development from P6 to P23. This indicates that, due to the loss of retinal input, the methylation profiles for some voltage-gated calcium channel activity genes cannot be established properly during eye opening. For instance, aberrant DNA methylation in Math5KO mice was identified in calcium voltage-gated channel subunits including CACNA1A, CACNA1C and CACNA1E. In addition, their expression levels are higher in Math5KO compared with WT dLGN (Fig. 4d, e and Additional file 2: Fig. S9D–F). Notably, CACNA1A gene codes for the alpha 1A subunit of the voltage-gated P/Q-type calcium channel (Cav2.1) and the mutations in CACNA1A gene lead to Episodic Ataxia type 2 (EA2) disorder with specific deficits in memory, executive functions and visual abilities. Although the connection between CACNA1E gene and visual experience has not been reported, mutations in CACNA1C gene have been associated with facial emotion recognition [37].

### Predicted epigenetic regulatory factors underlying dLGN development and in response to retinal input

**Fig. 5:**
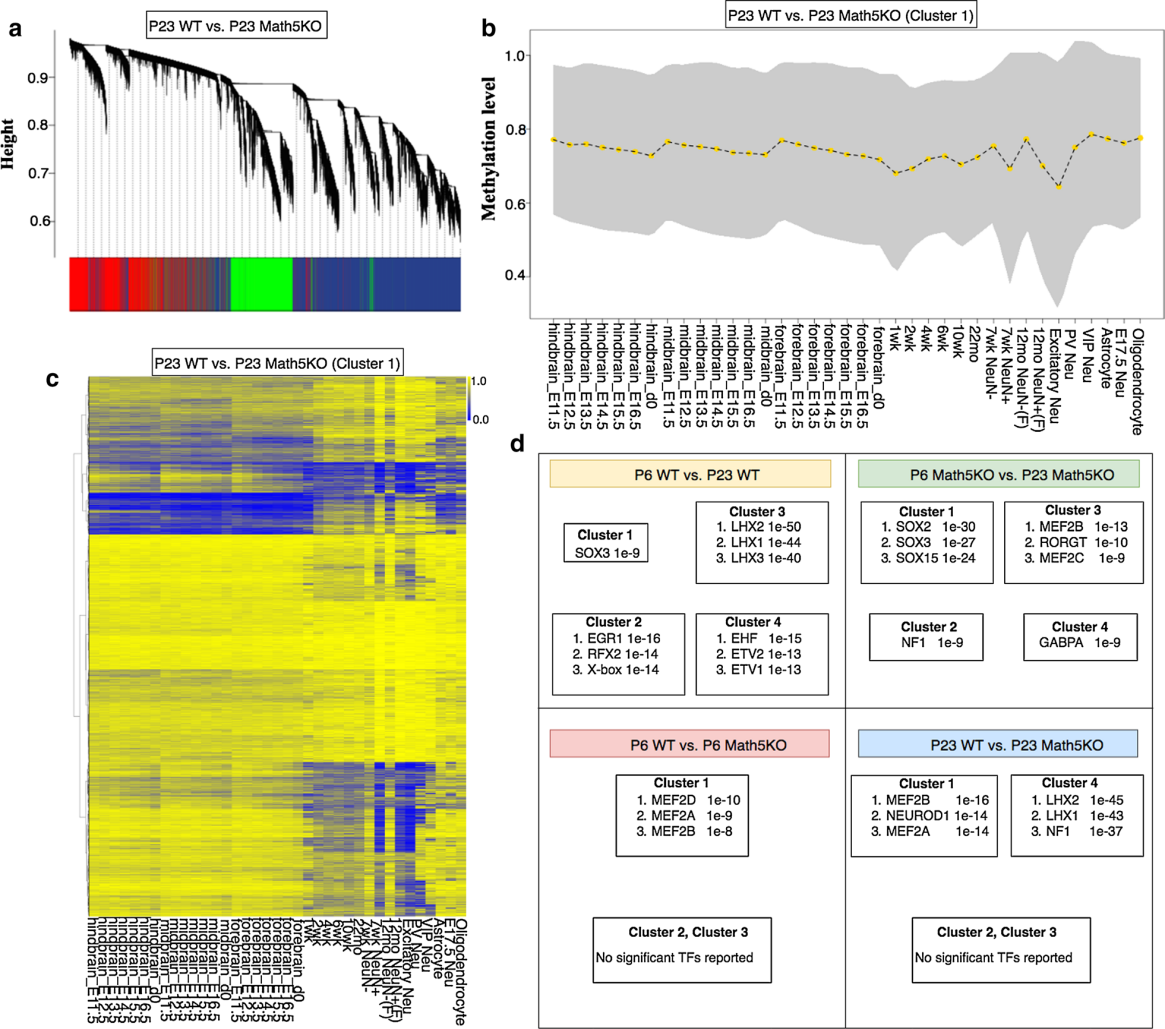
TF enrichment in different DMS clusters for four pairwise comparisons. **a** WGCNA clustering result for DMS sites identified in the comparison between P23 WT and P23 Math5KO. **b** Methylation profile overview of genomic regions with DMS sites in the first WGCNA cluster identified in the comparison between P23 WT and P23 Math5KO. Dotted line represents the average methylation level, and the shaded area represents the standard deviation of methylation levels. **c** Heat map of the methylation levels for genomic regions with DMS sites in the first WGCNA cluster identified in the comparison between P23 WT and P23 Math5KO. **d** Summarization of TF motif enrichments in clusters for four pairwise comparisons

To explore the regulatory mechanisms underlying dLGN methylation changes, we performed co-methylation and co-regulation analysis using a pipeline recently implemented in house [38]. We assumed that some genomic loci may be co-regulated by a common set of TFs if they share similar methylation profiles during development and across cell types. To identify co-methylated clusters, we first collected a total number of 63 mouse brain-related methylomes (Additional file 5: Table S4) and adopted the weighted correlation network analysis (WGCNA) R package [39] to group differentially methylated sites based on their methylation correlations across methylomes (Fig. 5a). The methylomes exploited in our analytic procedure comprise mostly development-related such as the methylomes for forebrain, midbrain and hindbrain regions at late gestation stages and cell-type-related methylomes including astrocytes, oligodendrocytes, excitatory neurons, PV neurons and VIP neurons. For all four pairwise comparisons, WGCNA grouped corresponding DMRs into only one cluster with a few that cannot be assigned to the co-methylated module. We did not identify any motif significantly enriched in DMRs for three pairwise comparisons: “P6 WT versus P23 WT,” “P6 WT versus P6 Math5KO” or “P23 WT versus P23 Math5KO.” Sox and Lhx family members were found to be with motifs enriched in DMRs of “P6 Math5KO versus P23 Math5KO.” We further extended analysis to DMSs and were able to determine three to four co-methylated clusters for each DMS list (Fig. 5b). Not surprisingly, these clusters show distinct methylation profiles during development and with brain cell-type-specific methylation patterns.

We next sought to identify transcription factors associated with methylation changes and observed that distinct sets of transcription factor binding motifs were enriched in genomic sequences for corresponding co-methylated clusters (Fig. 5c). Such an analysis identified two TF families, *EGR* and *MEF2,* that appeared potentially linked to excitatory neuron development in dLGN. They are among those top TFs enriched in co-methylated clusters that show decreased methylation during brain development and are with the lowest methylation level in excitatory neurons among all brain cell types (Additional file 2: Fig. S10–S13). Both EGR and MEF2 family members are transcription factors coordinating activity-dependent gene expression in neurons required for synapse formation, refinement and maturation [40]. EGR1 may be involved in the epigenetic programming of excitatory neurons in dLGN from P6 to P23 with an enrichment *p* value as 1e−16. Interestingly, for Math5KO mice, the significance for EGR1 enrichment at these ages is dramatically less at *p* value around 1e−2. This suggests that EGR1-mediated epigenetic programming in dLGN could be weakened due to the lack of retinal input.

**Fig. 6:**
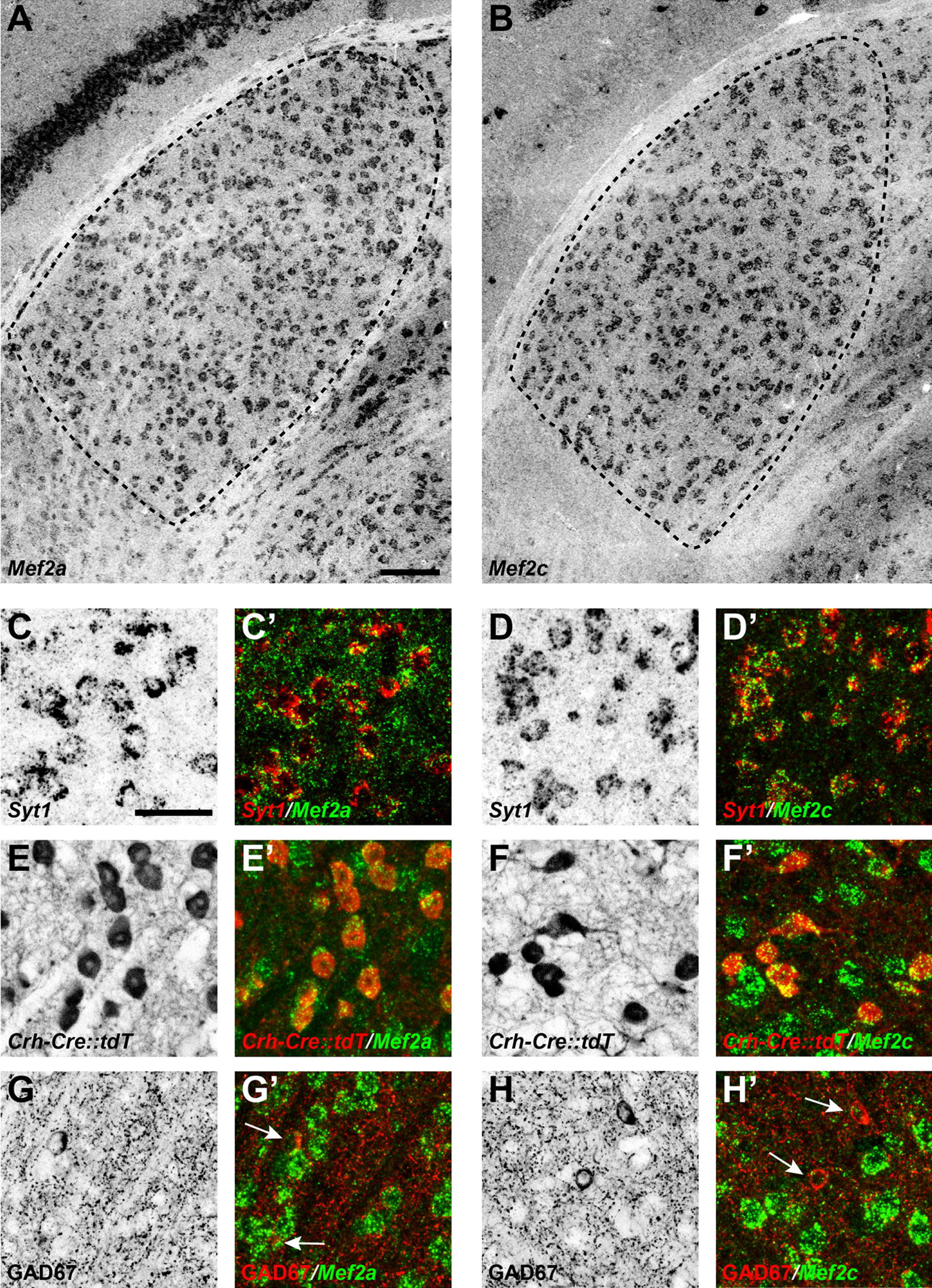
Expression of *Mef2a* and *Mef2c* mRNAs by relay cells in dLGN. ISH for *Mef2a* (**A**) and *Mef2c* (**B**) mRNAs in the dLGN of P25 wild type mice. dLGN encircled by black dots. Scale bar 100 µm. Double in situ hybridization (ISH) for *Syt1* and either *Mef2a* (**C**) or *Mef2c* (**D**) in P25 wild-type dLGN. ISH for either *Mef2a* (**E**) or *Mef2c* (**F**) in dLGN of P25 *Crh*-*Cre::tdT* mice. ISH for *Mef2a* (**G**) or *Mef2c* (**H**) and immunohistochemistry (IHC) for the inhibitory interneurons marker, GAD67, in P25 wild-type dLGN. White arrows depict GAD67 + interneurons. Scale bar, 40 µm (**C**–**H**)

MEF2 is the only TF identified with significant *p* value (less than 1e−10) associated with retinal-input-related methylation aberrations. In the DMS list identified from the comparison of WT P6 versus Math5 P6, the *p* values for all four MEF2 family members MEF2A/B/C/D are in the range from 1e−7 to 1e−10. In the DMS list identified from the comparison of WT P23 versus Math5 P23, the significance of motif enrichment for all four members increased. Thus, MEF2 may be required for methylation dynamics in early dLGN development. The lack of retinal input may compromise MEF2 function prior to the postnatal stage P6 and continues to have an impact up to P23. Of course, for this to be the case we hypothesized that MEF2 family members must be expressed by retino-recipient thalamocortical relay neurons in the developing dLGN. Our previous RNAseq analysis of the developing dLGN revealed that three MEF2 family members (MEF2A, MEF2C and MEF2D) are expressed in dLGN around eye opening [10]. To test the cell-specific expression of MEF2 family members in the developing dLGN, we generated riboprobes against MEF2A and MEF2C, the two family members whose expression in dLGN before and after eye opening appeared highest. In situ hybridization (ISH) with these riboprobes revealed robust *Mef2a* and *Mef2c* mRNA expression in dLGN compared with adjacent regions of the ventral thalamus, the overlying optic tract, or *stratum oriens* of the hippocampus (Fig. 6A, B). To assess whether *Mef2a*- or *Mef2c*-expressing cells were neurons we performed double ISH with riboprobes against *Syt1*, the gene encoding Synaptotagmin 1 (Syt1); all *Mef2a*- or *Mef2c*-positive cells contained Syt1 mRNA indicating neuron-specific expression of these transcription factors (Fig. 6C, D). Since two classes of neurons in exist in dLGN, we next assessed *Mef2a* and *Mef2c* expression in a transgenic reporter line in which excitatory thalamocortical relay neurons were fluorescently labeled (i.e., *Crh*-*Cre::Rosa*-*Stop*-*tdT*) and in wild-type mice in which inhibitory interneurons were immunolabeled with antibodies against Glutamate Decarboxylase 67 (GAD67), the enzyme required to convert glutamate into GABA (Fig. 6E–H). Our results revealed excitatory thalamocortical relay neurons, but not GABAergic interneurons, express MEF2 transcription factors in the developing visual thalamus. The expression of MEF2 family members by retino-recipient thalamocortical relay neurons supports the notion that retinal input may influence the expression and function of these transcription factors.

## Discussion

Genetic and environmental factors are two key drivers promoting epigenetic changes to allow cells with the same genetic content to express different sets of genes. During differentiation, unique methylation patterns were established in tissues to allow tissue-specific gene expression. In response to environment stimuli, cells convert external inputs into intracellular signals to initiate epigenetic modifications. In this study, we used Math5KO mice that lack retinal inputs to explore dLGN epigenetic dynamics before and after eye opening.

Although we cannot completely rule out the possibility that the methylation changes in Math5KO dLGN could be indirect side effects of the loss of Math5, several interesting observations were made in this study. First, we found that the development-related and retinal-input-induced epigenetic changes in dLGN show striking differences in genome distribution. Development-related DMSs are enriched on the 5′-UTR, CGI shore and CGI shelf regions and tend to co-localize together. On the other hand, retinal-input-induced DMSs are enriched on LTR repeats which may have unexpected functional relevance with dLGN development. Second, we observed that development-related DMSs are enriched in all types of enhancers identified in neurons, but retinal-input-induced DMSs are enriched in the centers of poised enhancers or enhancers with increasing H3K27ac marks in neurons stimulated by KCl. Notably, dLGN is a tissue composed of numerous cell types, while the enhancer landscapes were generated with primary neuron culture in vitro. Although the tissue and the cultured cells may not share exactly same histone landscape, our integrative analyses suggest the enhancers identified in neurons could be associated with methylation dynamics in dLGN. Third, besides genes with GO terms as “nervous system development,” we found that a number of transcription factors and voltage-gated calcium channel genes are epigenetically regulated during dLGN development and in response to retinal input. In addition, the integrative analysis with brain cell-type-specific methylation loci suggests that the expansion of glial cells in dLGN from P6 to P23 could explain the demethylation observed on the regulatory regions critical for gliogenesis.

In this study, we predicted that MEF2 and EGR family members might serve as important epigenetic regulators for dLGN development during eye opening. EGR family members are activity-dependent transcription factors whose expression increases within minutes following neuronal activation [41, 42]. Our recent study revealed that EGR1 is able to recruit DNA demethylation enzyme TET1 to demethylated EGR1 binding sites in neurons (data not shown). MEF2, or myocyte enhancer factor 2, is a family of four proteins (MEF2A/B/C/D) that play significant roles in the embryonic development of many tissues, including the developing cardiovascular and nervous systems [43]. In the developing brain and nervous system, MEF2 expression increases in newborn neurons and is required for the calcium-dependent survival of neurons promoted via neuronal activity [44]. With roles in regulating synaptic plasticity, in controlling spine numbers and in contributing to memory formation [45–47], MEF2 may interact with histone modification enzymes and chromatin-remodeling factors to determine the chromatin architecture in excitatory neurons [48, 49]. We observed that the motifs of MEF2 family members are enriched in the DMS lists between wild type and Math5KO at both P6 and P23. Such findings suggest that the loss of retinal input may have epigenetic impact prior to eye opening and such epigenetic aberrations continue to influence dLGN development and function after eye opening. Further experiments are desired to elucidate how MEF2 and EGR family members may participate in the epigenetic regulation of dLGN development.

## Materials and methods

### Animal care and dLGN sample collection

Wild-type C57BL/6 mice were obtained from Charles River (Wilmington, MA) or Harlan (Indianapolis, IN). *Math5* knockout (stock# 042298-UCD) and *Crh*-*Cre* (stock # 030850-UCD) mice were obtained from Wang et al. [24] and W. Guido (University of Louisville), respectively. P6 and P23 *Math5* knockout mutant and control mice were anesthetized by intraperitoneal injection of avertin (200 mg/kg), and their brain were dissected and coronally sectioned (400 µm) on a vibratome (Microm HM 650 V, Thermo Scientific). dLGN tissues were microdissected in ice-cold PBS under a dissection microscope. dLGN tissues can be easily identified by the optic tract and other neuropils that separate it from other regions of the thalamus. dLGN tissues were pooled from 8 (P6) or 4–6 (P23) mice and rapidly frozen in liquid nitrogen and kept in − 80 °C until DNA isolation. Four–five replicates were prepared for each group of mice. Mice were housed in a 12 h dark/light cycle and had ad libitum access to food and water. All experiments were performed in compliance with National Institutes of Health (NIH) guidelines and protocols and were approved by the Virginia Polytechnic Institute and State University IACUC.

### WGBS library construction and sequencing

Genomic DNA was extracted from mouse dLGN tissues using DNeasy mini Kit (Qiagen) according to the manufacturer’s instructions. One microgram genomic DNA per sample was spiked with 0.02% unmethylated cl857 Sam7 Lambda DNA (Promega) for library construction and sonicated to 200-bp fragments with Covaris M2 (AB). After end repair, dA tailing, the DNA fragments were ligated with cytosine-methylated Illumina TruSeq DNA adapters using T4 DNA ligase (NEB) overnight. After purification, adapter-ligated DNA fragments were subject to bisulfite conversion using the EpiTect Bisulfite Kit (Qiagen). After bisulfite conversion, the single-stranded uracil-containing DNA was subjected to 12 cycles of PCR amplification with Illumina TruSeq PCR primers and 2.5 U Pfu TurboCx Hotstart DNA polymerase (Agilent) to recover enough DNA for sequencing. The recovered libraries were sequenced on Hiseq 4000 platform (Illumina).

### WGBS-seq data analysis

Low sequencing quality bases and illumina sequencing adaptors were trimmed using Trim_Galore with the following parameters: Trim_galore -q 28 –illumina –length 30. After trimming, sequencing data were aligned to mm10 mouse genome using Bismark and Bowtie2. Using the modules embedding in Bismark, PCR duplicated read pairs were removed and the methylation information at both CpG and CH (H = A, C, or T) was extracted.

Fisher exact test was used to evaluate the significance of differential methylation on CpG site. Briefly, a contingency table for each of CpG sites was constructed based on the rows indicated two conditions and the columns indicated the number of methylated cytosines and unmethylated cytosines. In the test, CpG sites were required to have at least 10X reads covered. In order to control FDR, a total of 1000 permutations were performed for each CpG site with a sequential permutation method [50]. The number of true null hypotheses (m0) was estimated by a histogram method [26]. Based on the estimated m0, differentially methylated sites (DMSs) with adjusted *p* value less than or equal to 0.05 were identified. To determine differentially methylated regions (DMRs), we followed a two-step approach: Firstly, any two adjacent DMSs with at most 500 bp distance were merged into a cluster. We further selected clusters with at least 4 DMSs, and at least 80% of these DMSs shared the same direction in methylation changes, prone to be methylated or unmethylated. Secondly, DMRs were further identified from candidate clusters in which at least 80% of CpG sites shared the same direction in methylation changes and with a minimum methylation change as 0.1.

To identify cell-type-specific methylated CpG sites, we downloaded methylome data for five cell types from GEO database: Oligodendrocyte and astrocyte were downloaded from GSE89118, while excitatory neuron, PV neuron and VIP neuron were downloaded from GSE63137. Ten pairwise comparisons were made to identify differentially methylated CpG sites following aforementioned procedure. The cell-type-specific DMSs were defined as the CpG sites showing significant hyper- or hypomethylation pattern in a given cell type for at least 75% of the pairwise comparisons.

### RNAseq data generation and analysis

RNA from wild type and Math5KO dLGN was purified from pooled dLGN samples from four different ages (P3, P7, P14 and P23) as previously described [10]. RNAseq libraries were generated by Novogene Inc. and sequenced on Hiseq 4000 platform with 150 bp paired end mode (Illumina). After trimming bases of low quality and removing adapters, reads were mapped to mm10 by RSEM [51] with Bowtie2. The raw counts were employed to identify differentially expression genes by CORNAS [52]. The definition of differentially expression genes includes two requirements: (1) The alpha-value cutoff is 0.99, and (2) the fold change is equal to or greater than 1.5. The visualized data normalized to 1 million were generated by Bedtools [53].

### Analyses of transcriptional networks associated with methylation changes

A binomial test was employed to determine the significance of each of TFs on DMSs. The significance of the probability of DMSs overlapped with each of TFs over all DMSs was determined using a control set of CpG sites which were covered by at least 10X reads but not differentially methylated. The following steps were taken prior to the identification of enriched TF motifs: First, we selected DMS localized within − 10 kb from transcription start site (TSS) to transcription end site (TES). Next, we merged neighboring DMS within 200 bp range. Additionally, we removed all DMS with missing data in any of methylomes and provided a summary of DMS statistics (Additional file 6: Table S5A).

We used WGCNA R package [39] to group each DMS set into different clusters by calculating correlation patterns among DMS sites across methylome samples. When applied on a very large matrix r, WGCNA automatically divides the large matrix into smaller blocks and performs a two-level clustering. In the first step, DMS are pre-clustered into different blocks using a crude clustering technique. Next, for each block, it performs a network analysis by identifying clusters of highly correlated DMS and estimating cluster eigen-DMS. Finally, clusters whose eigen-DMS is highly correlated are merged. Thus, blockwise network analysis significantly reduces the memory footprint and the computational complexity. Results of WGCNA clustering are provided in Additional file 6: Table S5B.

We next carried out motif enrichment analysis of known transcription factors within each cluster for each DMS set using HOMER [54]. Since HOMER is a differential motif discovery algorithm, it requires two sets of sequences and then identifies the regulatory elements that are specifically enriched in on set (target set) relative to the other (background set). To create background sequences, for each DMS set we selected upstream and downstream regions situated ± 2 kb away from target regions. Thus, the number of background regions is double for each target set of target regions. As recommended by HOMER, the *p* value cutoff for significantly enriched motifs was set as 1e−10. The transcription factors that met this criterion were shown in this study, including their family members sharing similar motifs and close *p* values.

### Immunohistochemistry (IHC)

Mice were perfused with 4% paraformaldehyde (in PBS), and their brains were dissected and kept at 4 °C in 4% PFA overnight. Brains were transferred to 30% sucrose solution and kept at 4 °C for 2–3 days before being embedded in Tissue Freezing Medium (Electron Microscopy Sciences, Hatfield, PA). Cryosectioned (16 μm) brains were incubated in blocking buffer (2.5% bovine serum albumin, 5% normal goat serum, 0.1% Triton-X in PBS) for 1 h. GAD67 primary antibody (Millipore MAB5406) was diluted (1:700) in blocking buffer and incubated on tissue sections for > 12 h at 4 °C. Sections were incubated with anti-mouse secondary antibody (Invitrogen Life Technologies; 1:1000 in blocking buffer) for 1–2 h at room temperature. Images were acquired on a Zeiss LSM 700 confocal microscope.

### In situ RNA hybridization (ISH)

*Mef2a* (clone ID 4979487), *Mef2c* (clone ID 4500786) and *Syt1* (clone ID 5363062) cDNAs were obtained from GE Dharmacon. Riboprobes against *Mef2a*, *Mef2c* and *Syt1* mRNAs were generated as described previously [10]. ISH was performed on 16 μm PFA-perfused coronally cryosectioned brain tissue prepared as described above. Tissues were prepared and hybridized at 60 °C as previously described [10]. Bound riboprobes were detected by either horseradish peroxidase (POD)-conjugated anti-DIG or anti-fluorescent antibodies (Roche #: 11426346910 and 11207733910), followed by Tyramide Signal Amplification systems (PerkinElmer #: NEL75300 1KT). Slides were visualized on a Zeiss LSM 700 confocal microscope.

## Additional files

**Additional file 1: Table S1.** Summary statistics for four dLGN WGBS libraries.

**Additional file 2: Fig. S1.** Distribution of read depth for CpG sites determined in four dLGN WGBS libraries. **Fig. S2.** Distribution of CpG methylation levels determined for four dLGN WGBS libraries. **Fig. S3.** Venn diagram of DMS lists identified from four pairwise comparisons. **Fig. S4.** Relationships between mCH and gene expression. The mCH profiles for (A) P6 WT, (B) P6 Math5KO, (C) P23 WT and (D) P23 Math5KO. Red line denote the group of genes with the top one-third expression; green line denote the group of genes with the median one-third expression; blue line denote the group of genes with the bottom one-third expression; and black line show the group of genes not expressed. The average expression levels at P3 and P7 were shown for P6. **Fig. S5.** Pairwise comparisons identified common sets of 463 upregulated (A) and 554 downregulated (B) genes from P3 to P23 were identified in both WT and Math5KO. No gene was identified to be overlapped for upregulated (**C**) or downregulated (**D**) in Math5KO in pairwise comparisons between WT and Math5KO dLGN across all four time points. **Fig. S6.** The mCH profiles for 463 upregulated (Green) and 554 downregulated (Red) genes from P3 to P23 in P7 WT (A), P7 Math5KO (B), P23 WT (C) and P23 Math5KO methylomes. **Fig. S7.** Heat map (A) and scatter plot (B) for gene expression profiles of 61 genes which promoters overlapped with DMRs showing methylation increased in P23 Math5KO. Heat map was generated using RNAseq data from four time points with color bar showing log (1 + TPM), and scatter plot was generated with RNAseq data at P23 for WT and Math5KO with X- and Y-axis showing log (1 + TPM**). Fig. S8.** Methylation profiles of DMRs during mouse brain development. **Fig. S9.** DNA Methylation for DMRs and gene expression profiles for Lhx2 and CACNA1E loci. **Fig. S10.** WGCNA clustering and motif enrichment analysis for DMS sites identified in the comparison between P6 WT and P23 WT. (A) WGCNA clustering. (B) Methylation profiles of different clusters. (C) Top TFs with motifs significantly enriched in each cluster predicted by HOMER. **Fig. S11.** WGCNA clustering and motif enrichment analysis for DMS sites identified in the comparison between P6 Math5KO and P23 Math5KO. (A) WGCNA clustering. (B) Methylation profiles of different clusters. (C) Top TFs with motifs significantly enriched in each cluster predicted by HOMER. **Fig. S12.** WGCNA clustering and motif enrichment analysis for DMS sites identified in the comparison between P6 WT and P6 Math5KO. (A) WGCNA clustering. (B) Methylation profiles of different clusters. (C) Top TFs with motifs significantly enriched in each cluster predicted by HOMER. **Fig. S13.** WGCNA clustering and motif enrichment analysis for DMS sites identified in the comparison between P23 WT and P23 Math5KO. (A) WGCNA clustering. (B) Methylation profiles of different clusters. (C) Top TFs with motifs significantly enriched in each cluster predicted by HOMER.

**Additional file 3: Table S2.** Summary of eight dLGN RNAseq libraries and differentially expressed genes.

**Additional file 4: Table S3.** Summary of DMRs and GO enrichment analysis for genes associated with DMRs.

**Additional file 5: Table S4.** Summary of “omics” dataset included in this study.

**Additional file 6: Table S5.** Summary of WGCNA clustering results.
