# Supplementary material for: Retinal-input-induced epigenetic dynamics in the developing mouse dorsal lateral geniculate nucleus
Source: Epigenetics Chromatin. 2019 Feb 14;12:13. doi: 10.1186/s13072-019-0257-x (PMC6374911; doi:10.1186/s13072-019-0257-x)

**Additional file Information**

**Retinal Input Induced Epigenetic Dynamics in the Developing Mouse Dorsal Lateral Geniculate Nucleus**

**Contents:**

Additional Tables (Table S1-S5)

Additional Figures S1-S12

**Additional Tables:**

**Table S1. Summary statistics for four dLGN WGBS libraries.** (Excel file: Table S1)

**Table S2. Summary of eight dLGN RNAseq libraries and differentially expressed genes.** (Excel file: Table S2)

**Table S3. Summary of DMRs and GO enrichment analysis for genes associated with DMRs.** (Excel file: Table S3)

**Table S4. Summary of “omics” dataset included in this study** (Excel file: Table S4)

**Table S5. Summary of WGCNA clustering results.** (Excel file: Table S5)

**Figure S1 Distribution of read depth for CpG sites determined in four dLGN WGBS libraries**

**Figure S2 Distribution of CpG methylation levels determined for four dLGN WGBS libraries**

**Figure S3 Venn diagram of DMS lists identified from four pairwise comparisons**

**Figure S4 Relationships between mCH and gene expression.** The mCH profiles for (A) P6 WT, (B) P6 Math5KO, (C) P23 WT and (D) P23 Math5KO. Red line denotes the group of genes with the top one-third expression; green line denotes the group of genes with the median one-third expression; blue line denotes the group of genes with the bottom one-third expression; and black line shows the group of genes not expressed. The average expression levels at P3 and P7 were shown for P6.

**Figure S5 Pairwise comparisons identified common sets of 463 upregulated (A) and 554 downregulated (B) genes from P3 to P23 were identified in both WT and Math5KO.** No gene was identified to be overlapped for upregulated (**C**) or downregulated (**D**) in Math5KO in pairwise comparisons between WT and Math5KO dLGN across all four time points.

**Figure S6** The mCH profiles for 463 upregulated (Green) and 554 downregulated (Red) genes from P3 to P23 in P7 WT (A), P7 Math5KO (B), P23 WT (C) and P23 Math5KO methylomes.

**Figure S7** Heatmap (A) and scatter plot (B) for gene expression profiles of 61 genes which promoters overlapped with DMRs showing methylation increased in P23 Math5KO. Heatmap was generated using RNAseq data from four time points with color bar showing log (1+TPM) and scatter plot was generated with RNAseq data at P23 for WT and Math5KO with X- and Y-axis showing log (1+TPM).

**Figure S8** Methylation profiles of DMRs during mouse brain development

**Figure S9** DNA Methylation for DMRs and gene expression profiles for Lhx2 and Cacna1e loci


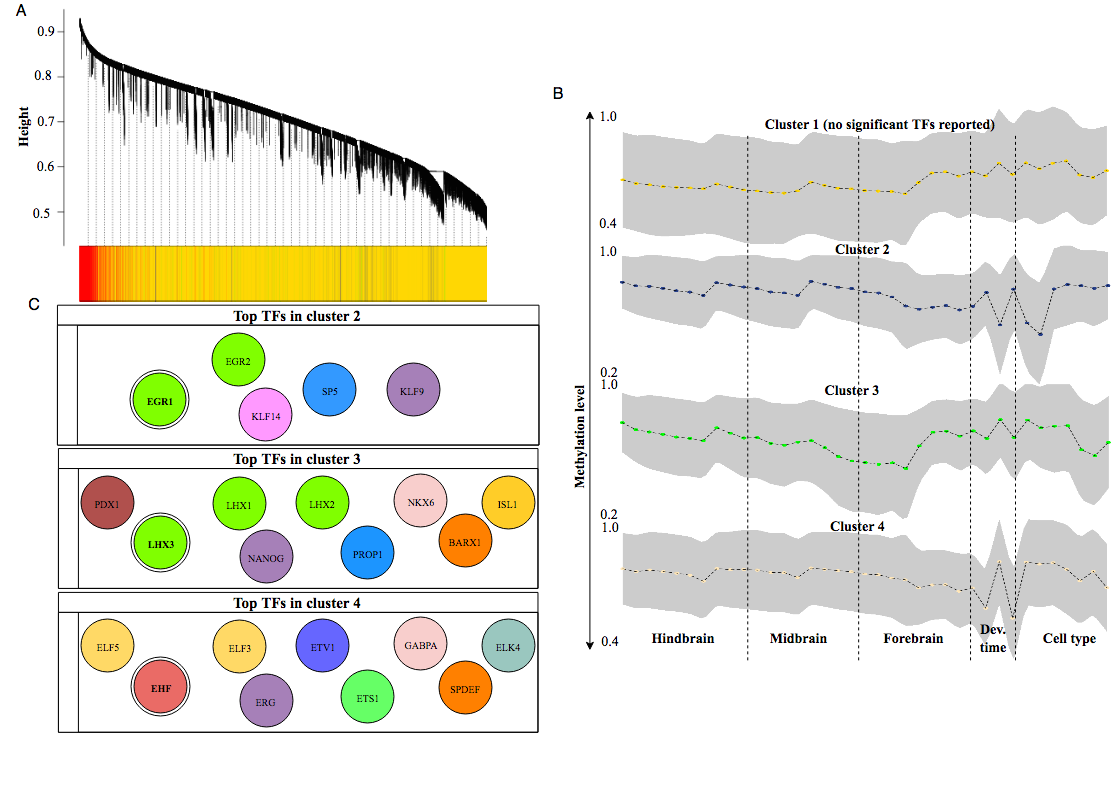


**Figure S10** WGCNA clustering and motif enrichment analysis for DMS sites identified in the comparison between P6 WT and P23 WT. (A) WGCNA clustering. (B) Methylation profiles of different clusters. (C) Top TFs with motifs significantly enriched in each cluster predicted by HOMER.

**Figure S11** WGCNA clustering and motif enrichment analysis for DMS sites identified in the comparison between P6 Math5KO and P23 Math5KO. (A) WGCNA clustering. (B) Methylation profiles of different clusters. (C) Top TFs with motifs significantly enriched in each cluster predicted by HOMER.


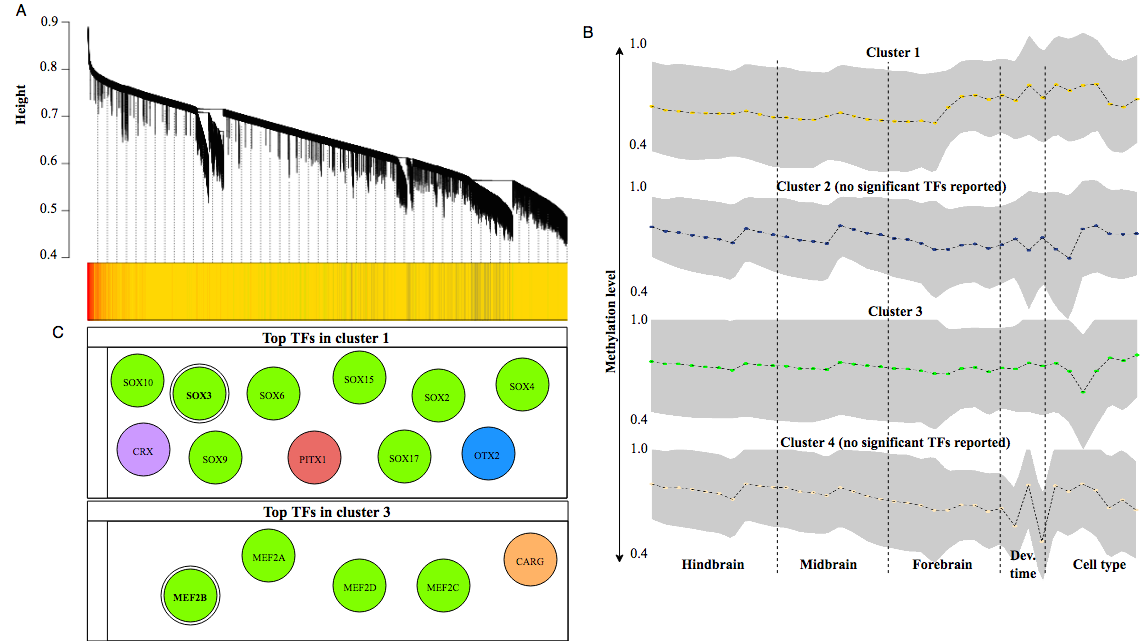


**Figure S12** WGCNA clustering and motif enrichment analysis for DMS sites identified in the comparison between P6 WT and P6 Math5KO. (A) WGCNA clustering. (B) Methylation profiles of different clusters. (C) Top TFs with motifs significantly enriched in each cluster predicted by HOMER.


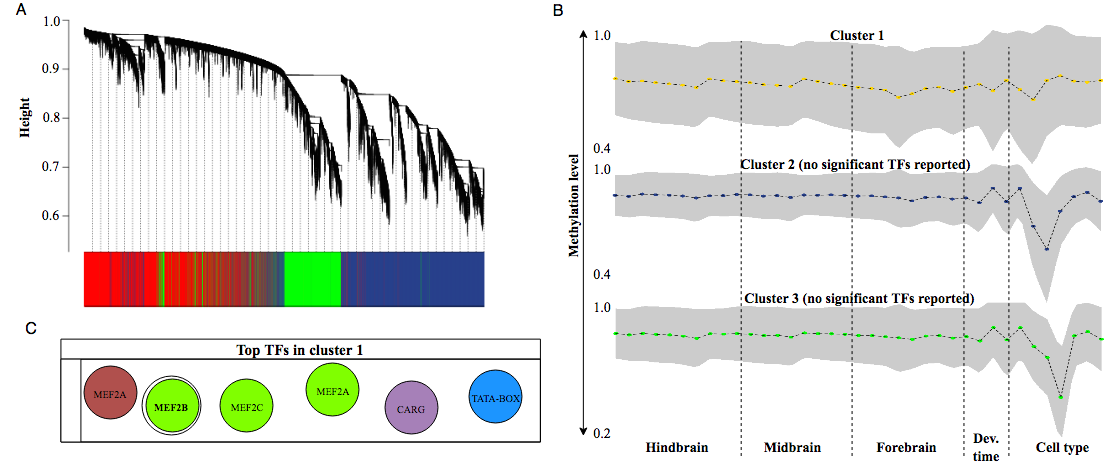


**Supplementary Figure 13** WGCNA clustering and motif enrichment analysis for DMS sites identified in the comparison between P23 WT and P23 Math5KO. (A) WGCNA clustering. (B) Methylation profiles of different clusters. (C) Top TFs with motifs significantly enriched in each cluster predicted by HOMER.


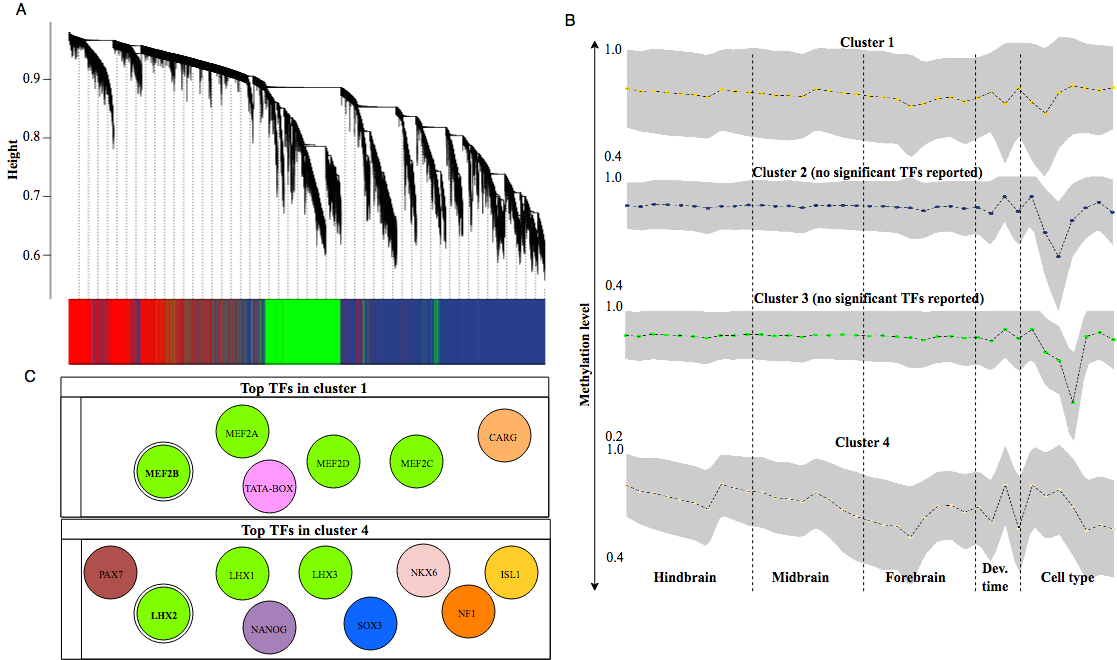

Supplement: Supplementary file 2 — Additional file 2: Fig. S1. Distribution of read depth for CpG sites determined in four dLGN WGBS libraries. Fig. S2. Distribution of CpG methylation levels determined for four dLGN WGBS libraries. Fig. S3. Venn diagram of DMS lists identified from four pairwise comparisons. Fig. S4. Relationships between mCH and gene expression. The mCH profiles for (A) P6 WT, (B) P6 Math5KO, (C) P23 WT and (D) P23 Math5KO. Red line denote the group of genes with the top one-third expression; green line denote the group of genes with the median one-third expression; blue line denote the group of genes with the bottom one-third expression; and black line show the group of genes not expressed. The average expression levels at P3 and P7 were shown for P6. Fig. S5. Pairwise comparisons identified common sets of 463 upregulated (A) and 554 downregulated (B) genes from P3 to P23 were identified in both WT and Math5KO. No gene was identified to be overlapped for upregulated (C) or downregulated (D) in Math5KO in pairwise comparisons between WT and Math5KO dLGN across all four time points. Fig. S6. The mCH profiles for 463 upregulated (Green) and 554 downregulated (Red) genes from P3 to P23 in P7 WT (A), P7 Math5KO (B), P23 WT (C) and P23 Math5KO methylomes. Fig. S7. Heat map (A) and scatter plot (B) for gene expression profiles of 61 genes which promoters overlapped with DMRs showing methylation increased in P23 Math5KO. Heat map was generated using RNAseq data from four time points with color bar showing log (1 + TPM), and scatter plot was generated with RNAseq data at P23 for WT and Math5KO with X- and Y-axis showing log (1 + TPM). Fig. S8. Methylation profiles of DMRs during mouse brain development. Fig. S9. DNA Methylation for DMRs and gene expression profiles for Lhx2 and CACNA1E loci. Fig. S10. WGCNA clustering and motif enrichment analysis for DMS sites identified in the comparison between P6 WT and P23 WT. (A) WGCNA clustering. (B) Methylation profiles of different clusters. (C) T [file 13072_2019_257_MOESM2_ESM.docx]
